# Supplementary material for: Autophagy Mediates the Degradation of Plant ESCRT Component FREE1 in Response to Iron Deficiency
Source: Int J Mol Sci. 2021 Aug 16;22(16):8779. doi: 10.3390/ijms22168779 (PMC8396019; doi:10.3390/ijms22168779)
Supplement: Supplementary file 1 [file ijms-22-08779-s001.zip › ijms-1322389-supplementary.pdf]

**Table S1. Primers used in this article**

Primers used for qRT-PCR

| | |
|---|---|
| ATG4a-qPCR-F | GGCTGCATTGCAACTAGATTT |
| ATG4a-qPCR-R | TCAGAGCATTTGCCAGTCATCTTCAC |
| ATG4b-qPCR-F | CTTTCACGTTCCCTCAAAGC |
| ATG4b-qPCR-R | TTGCAATGGTAAGACGATGTG |
| ATG6-qPCR-F | GGAGATTCCAGATAAAAGTCGG |
| ATG6-qPCR-R | ACGATGGTAAGGGAGTGGTG |
| ATG8a-qPCR-F | CAATTTGTATACGTGGTTCGT |
| ATG8a-qPCR-R | AGCAACGGTAAGAGATCCAA |
| ATG8b-qPCR-F | TTGGCCAATTTGTGTACGTT |
| ATG8b-qPCR-R | TCCACCAAATGTGTTCTCTCC |
| UBQ10-qPCR-F | CGGAAAGCAGTTGGAGGATGG |
| UBQ10-qPCR-R | CGGAGCCTGAGAACAAGATGAAG |
| FREE1-qPCR-F | TATTACTCACCGTATGATCAGCATC |
| FREE1-qPCR-R | ATGAATAAGGCGCGGGATTAG |
